# Current interventional model for movement in Parkinson’s disease: network meta-analysis based on the improvement of motor ability

**DOI:** 10.3389/fnagi.2024.1431277

**Published:** 2024-09-10

**Authors:** Zhao HongFei, Zhang Li, Li Liang, Guo Wan Ru, Huang Lan Yi, Wang Zhen

**Affiliations:** ^1^Wushu College, Shanghai Sport University, Shanghai, China; ^2^Xiamen Medical College, Xiamen, China; ^3^School of Psychology, Shanghai Sport University, Shanghai, China

**Keywords:** network meta-analysis, Parkinson’s disease, exercise intervention, motor function, randomized controlled trial

## Abstract

**Aim:**

To identify optimally therapeutic exercise interventions for improving motor ability among patients with Parkinson’s disease (PD), we conducted a network meta-analysis (NMA) of randomized controlled trials comparing different exercise regimens.

**Methods:**

Relevant RCTs were retrieved by searching PubMed, Embase, Cochrane, Web of Science, CINAHL, CBM, China National Knowledge Infrastructure (CNKI), Wan fang, VIP, and other databases from inception to July 9, 2023 is available in English as the primary language. Exercise outcomes as measured by Movement Disorder Society- Unified Parkinson’s Disease Rating Scale Part III (MDS-UPDRS-III) score change were evaluated and ranked using STATA software version 18.0. All included studies were assessed for methodological quality using the Cochrane Risk of Bias tool.

**Results:**

The final NMA included 71 studies involving 3,732 participants, 87 intervention experiments, and 27distinct interventions. Although most exercise interventions showed some efficacy (reducing MDS-UPDRS-III score), cumulative ranking probability surface (SUCRA) values indicated that the best exercise interventions for motor function improvement were archery (95.6%), riding a bicycle (80.9%), and binary rhythm dance (80.8%).

**Conclusion:**

An exercise intervention comprising archery, cycling, and(or) binary rhythm dance may yield superior improvements in motor function among patients with Parkinson’s disease.

## 1 Introduction

Parkinson’s disease (PD) is a neurodegenerative disorder characterized neuropathologically by the degeneration of dopaminergic neurons and behaviorally by progressive deterioration of motor function and eventually of cognitive capacity. It is the second most common chronic neurodegenerative disease of the central nervous system after Alzheimer’s disease (AD). Like AD, PD onset risk increases with age, so as expected prevalence is rising globally due to population aging, and in particular the number and proportion of individuals aged 65 or older (Hirsch et al., 2016). Current projections suggest that the global prevalence of PD patients will exceed 12 million by the year 2040 (Dorsey and Bloem, 2018). In addition to the aged, a growing number of individuals under the age of 50 are being diagnosed with early-onset PD (Dorsey et al., 2018). The typical clinical manifestations of early- to intermediate-stage PD are bradykinesia, rigidity, reduced range of motion, and diminished automaticity (Mirelman et al., 2019), motor deficits that substantially reduce functional independence and quality of life.

The first-line treatment for PD is dopamine replacement therapy. While dopaminergic medications can improve motor functions such as walking speed and stride length, prolonged therapy often leads to motor fluctuations, dyskinesia (Albani et al., 2014), and non-motor symptoms like hallucinations and impulsive compulsive behaviors (Weintraub et al., 2022). Surgical interventions, such as deep brain stimulation (DBS) may also accelerate the initiation of motor responses and ameliorate functional disturbances, but electrode implantation carries risks of infection, rejection and poor or suboptimal stimulus targeting. Moreover, no current treatment can prevent or alleviate non-motor symptoms and disease progression.

The development of therapies that can augment or replace drug and surgical interventions is a major focus of current PD research. Such studies have reported that exercise, physical rehabilitation, psychological interventions, and caregiving can be cost-effective and feasible adjunct therapies with long-term adherence. In fact, cross-sectional, longitudinal observational, and prospective interventional trials support exercise therapy as more effective for addressing motor symptoms than current pharmacological approaches (Dutta et al., 2022; Foster et al., 2013; Gamborg et al., 2022; Schenkman et al., 2018). However, these improvements are not always observed, potentially due to variations in cognitive engagement, the severity of the disease, and the specific exercises used (Canning et al., 2012; Skidmore et al., 2008). In addition, inconsistencies in outcome may reflect the use of different evaluation metrics. In 2001, the Movement Disorder Society (MDS) commissioned a revision of the Unified Parkinson’s Disease Rating Scale (UPDRS) initially developed in the 1980s. This new version, called the MDS-UPDRS, was further revised in 2008 to enhance its assessment capabilities. It has since become a widely utilized clinical rating scale for comprehensively evaluating various symptoms and complications of PD (Goetz et al., 2007; Ramaker et al., 2002). Among other advantages, this widespread application of the MDS-UPDRS may improve consistency across studies and thereby enhance the feasibility of pooled data analyses (e.g., meta-analyses).

Network meta-analysis (NMA) is a versatile technique for simultaneously comparing multiple interventions (e.g., A vs. B, B vs. C) from individual studies (Lu and Ades, 2004). Moreover, by combining direct and indirect comparisons, NMA techniques can rank the relative efficacies of multiple interventions for selecting the optimal regimen (Garcia-Ruiz et al., 2014). In the current NMA, the MDS-UPDRS was selected as the outcome measure and various exercise interventions evaluated in randomized controlled trials (RCTs) were systematically ranked according to the improvement (decrease) in MDS-UPDRS score post-intervention. The first part of the MDS-UPDRS addresses “non-motor experiences of daily living,” the second “motor experiences of daily living,” the third part remains dedicated to “motor examination,” and the fourth part focuses on “motor complications.” The third part, MDS-UPDRS-III (motor examination) has demonstrated high reliability, validity, and sensitivity to change following treatment, with an assessment time of less than 15 min (Goetz et al., 2008). Therefore, MDS-UPDRS-III is particularly useful for evaluating the efficacies of specific exercise interventions. The exercise interventions examined in the current NMA include treadmill training, stretch training, aerobic exercise, aquatic exercise, balance and gait training, dual-task training, dance (e.g., tango, waltz, Irish dance, Sardinian dance, folk dance, different rhythm-based dance therapies), qigong practices (e.g., Eight Brocades, Five Animal Frolics, Six Healing Sounds), Tai Chi, mindfulness meditation, resistance exercises (e.g., weightlifting, resistance band exercises, progressive resistance exercise), exercise games, rock climbing deficits. While many systematic reviews and meta-analyses have promoted the effectiveness of various physical therapies in PD, most have included non-randomized controlled trials or lacked quantitative analysis. Moreover, these reviews often compared non-pharmacological, physical interventions with placebos, waitlists, or standard treatments, providing insufficient evidence for ranking by efficacy. Therefore, our objective is to systematically review previous RCTs, on diverse exercise interventions for PD, reevaluate the efficacy of each by pooled analysis, and rank exercises by efficacy to provide clinicians and patients with evidence-based selection criteria.

## 2 Methods

### 2.1 Eligibility criteria and literature search

PubMed, Embase, Cochrane, Web of Science, CINAHL, CBM, China National Knowledge Infrastructure (CNKI), Wanfang, VIP, and other databases were searched from inception to July 9, 2023, without language restrictions. Search strings included a combination of Medical Subject Headings (MeSH terms or Emtree terms) and free-text terms related to PD (“Parkinson’s disease”, “idiopathic Parkinson’s disease”, “Lewy body dementia”, “tremor paralysis”), exercise intervention (“aerobic exercise”, “strength training”, “balance exercise”, “balance”, “dual-task training”, “stretching exercise”, “Tai Chi”, “Five Animal Frolics”, “Eight Brocades”, “qigong”, “yoga”, “dance”, “boxing”, “resistance training”, “aquatic exercise”) and RCTs (“randomized controlled trial”, “random control”, “placebo”).The MeSH terms and free words were linked by “OR” within each group, and the groups were linked by “AND” for the search. Study selection, data gathering, and reporting were conducted in compliance with the Preferred Reporting Items for Systematic Reviews and Meta-analyses (PRISMA) statement and the Cochrane Collaboration extension statement (Hutton et al., 2015).

### 2.2 Study selection criteria

Inclusion criteria were based on the PICO (Participant, Intervention, Outcome, Study Design) guideline as follows: (1) Participants were early-to mid-stage Parkinson’s disease classified according to the Hoehn and Yahr (H&Y) scale (Stages I–III); (2) The Intervention was exercise training; (3) Outcome was change in MDS-UPDRS-III score; (4) Study Design was RCT available in English as the primary language.

Studies were excluded if participants had other neurological disorders, the primary outcome measure was not the MDS-UPDRS-III, there was no randomly selected control group, or if only a single acute training event was examined. In addition, feasibility, effectiveness, and pilot studies were excluded, as were study protocols.

**Figure 1:**
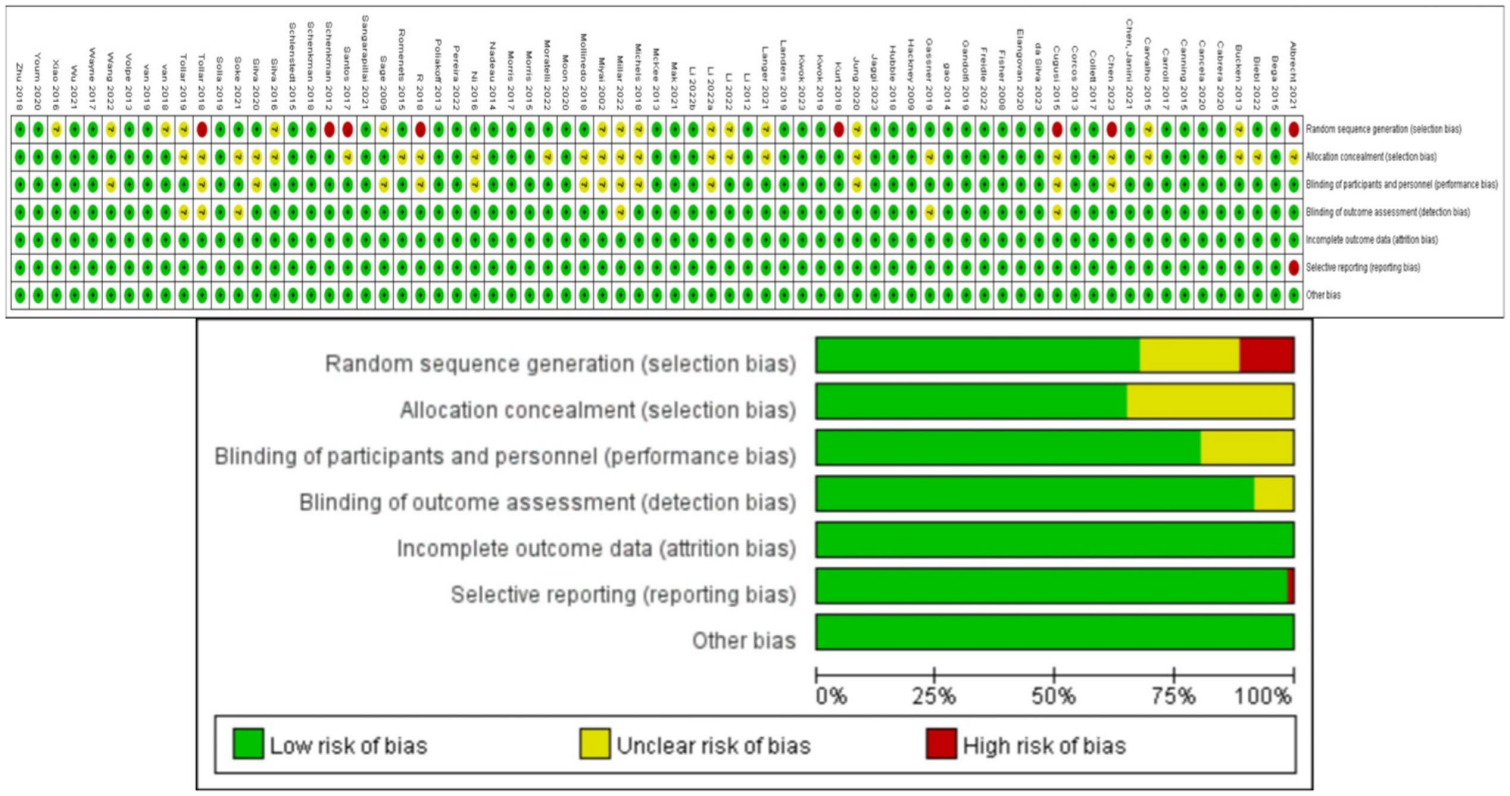
Analysis of the risk of bias in accordance with the Cochrane collaboration guideline.

Two authors (ZHF and ZL) independently screened titles, abstracts, and full texts for potential inclusion, and discussed disagreements until reaching a consensus. Data was extracted by the first author (ZHF), and extracted parameters included including participant characteristics (sample size, age, disease duration, Hoehn and Yahr stage, MDS-UPDRS-III scores at baseline and post-intervention), medication status (ON or OFF) during the trial, and type, frequency, and duration, of the exercise intervention. In addition to published RCTs retrieved from the aforementioned literature databases, reports on ongoing or upcoming trials were retrieved from the U.S. National Library of Medicine ClinicalTrials.gov and the Chinese Clinical Trial Registry. Grey literature was also considered. Finally, the reference lists of included articles were searched for eligible studies. Six potential sources of bias (risk of bias, RoB) were assessed for each RCT using the revised Cochrane Collaboration Tool (Abraha and Montedori, 2010): (1) bias from the randomization process, (2) deviation from intended interventions, (3) missing outcome data, (4) bias from the outcome measurement, (5) selective outcome reporting, and (6) overall bias. During this process, the first author (ZHF) independently screened the articles and any discrepancies were resolved by discussion with a third researcher (LL) until consensus was achieved. The risk for each primary source of bias was defined as either “low,” “medium,” or “high” for each trial, and a color-coded risk of bias table was constructed (Figure 1).The risk assessment for each trial was independently entered into Review Manager (RevMan 5.4), generating a summary of bias risk alongside the meta-analysis results.

## 3 Statistical analysis

**Figure 2:**
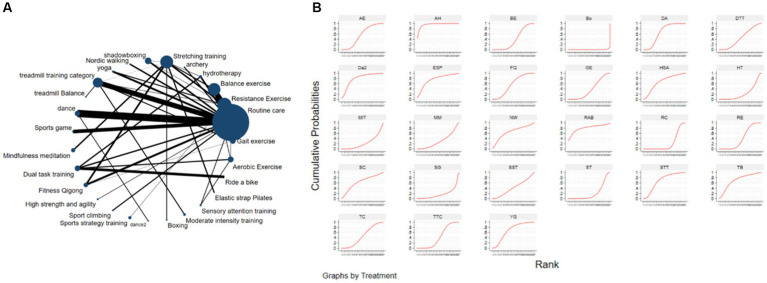
The NMA figure for MDS-UPDRS-III **(A)**. The SUCRA plot for MDS-UPDRS-III **(B)**. GE, Gait exercise; RC, Routine care; RE, Resistance Exercise; BE, Balance exercise; HT, hydrotherapy; AH, archery; ST, Stretching training; TC, shadowboxing; NW, Nordic walking; YG, yoga; TTC, treadmill training category; TB, treadmill Balance; DA, dance; SG, Sports game; MM, Mindfulness meditation; DTT, Dual task training; FQ, Fitness Qigong; HAS, High strength and agility; SC, Sport climbing; SST, Sports strategy training; Da2, dance2(Duality Rhythm Dance); Bo, Boxing; MIT, Moderate intensity training; STT, Sensory attention training; ESP, Elastic strap Pilates; RAB, Ride a bike; AE, Aerobic Exercise.

The NMA was conducted using STATA 18.0, and the Frequentist framework was employed following the PRISMA NMA guidelines. For all eligible RCTs, the post-intervention mean MDS-UPDRS-III score (with standard deviation) was retrieved for comparison across studies. To depict all available effects for each exercise intervention, a network evidence graph was generated as a concise summary (Figure 2A). In the network graph, nodes represent exercise interventions, node size is proportional to the total number of participants in the studies, the connecting edges between nodes indicate direct pairwise comparisons, and edge is indicative of effect magnitude.

The Surface Under the Cumulative RAnking (SUCRA) curve (Figure 2B) is a simple numerical statistic indicating the cumulative ranking probability for each intervention, and serves as an metric for grading the superiority or inferiority of exercise interventions (Page et al., 2016). Specifically, a larger SUCRA value indicates a greater likelihood that a particular exercise intervention is highly ranked (relatively more effective), while a lower value suggests that the intervention is likely less effective. We examined global consistency and employed the node-splitting model to assess local consistency. A *p* > 0.05 indicated no significant inconsistency between direct and indirect comparisons, and a consistency model was used. Otherwise, an inconsistency model was employed.

**Figure 3:**
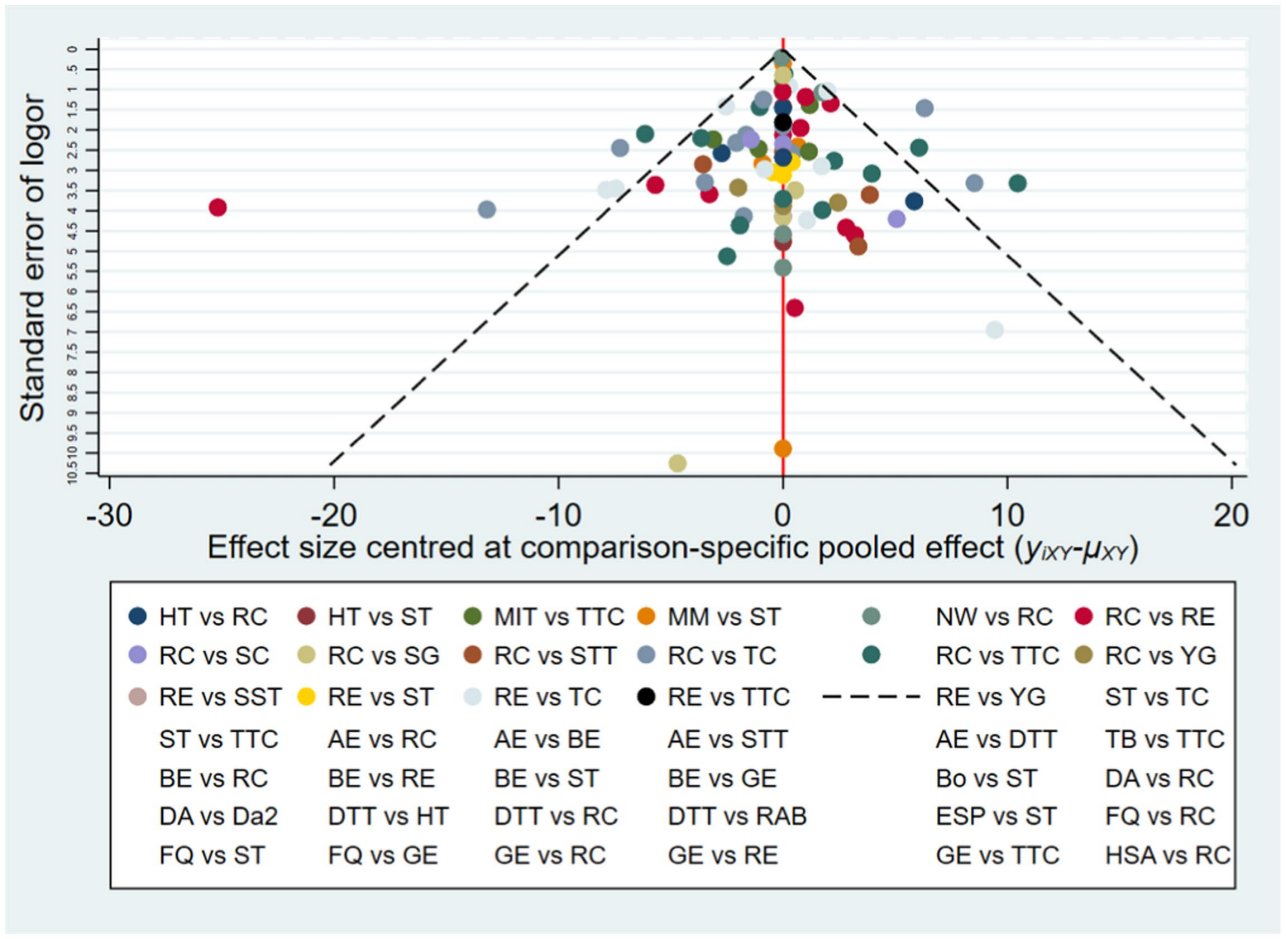
Funnel plot on publication bias of MDS-UPDRS-III.

To detect the presence of publication bias, selective reporting, and other biases, we constructed a funnel plot (Figure 3) (Egger et al., 1997). The funnel plot is a simple scatter plot that reflects the estimated intervention effect of a single study with a certain sample size or precision. The distribution width and symmetry are indicative of study heterogeneity and publication bias, respectively. The advantage of the funnel plot is that it is intuitive, as relative differences in effect size can be observed directly.

## 4 Results

### 4.1 Study identification

**Figure 4:**
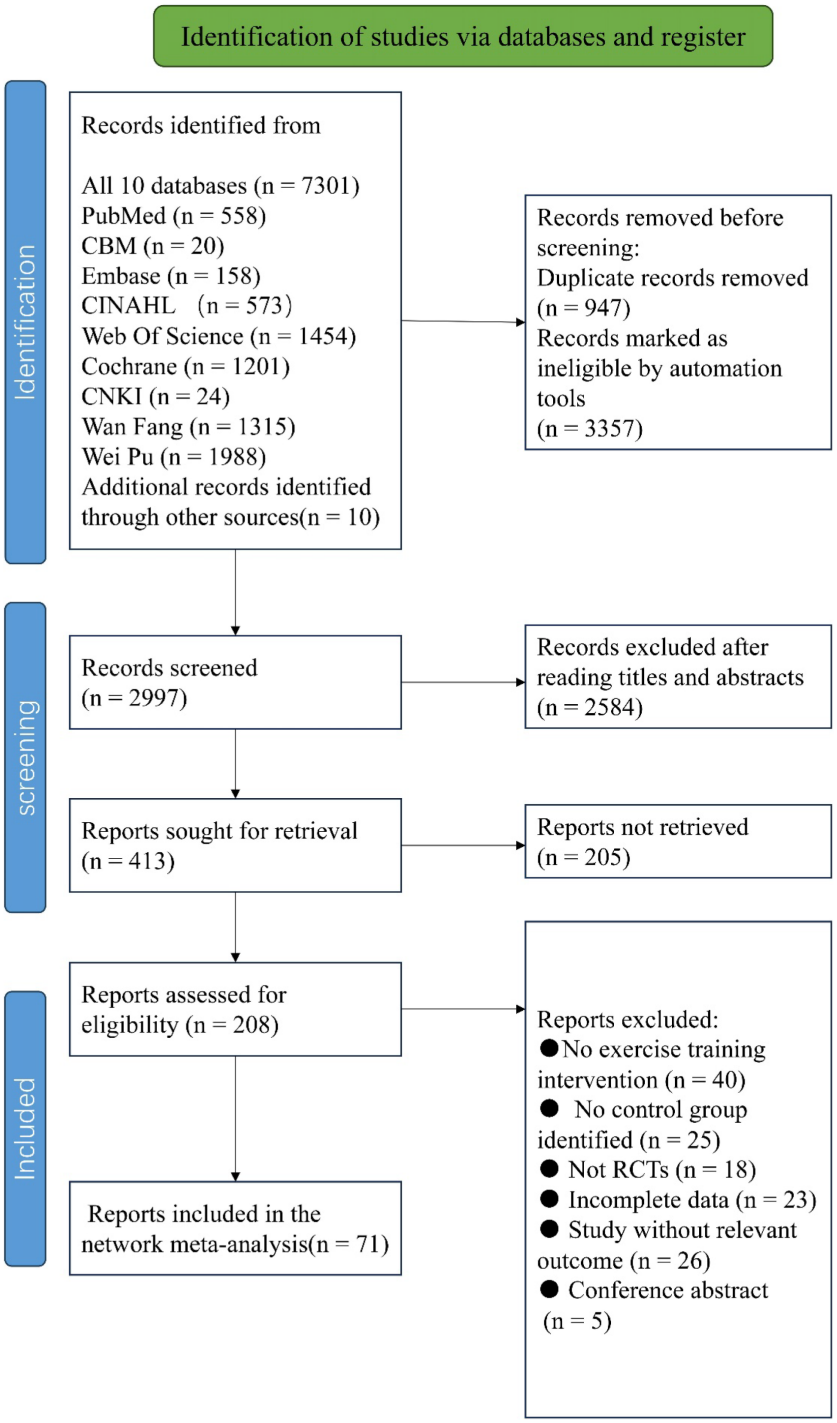
The process of selection of the eligible studies.

A total of 7,301 articles were retrieved using the pre-established search strategy. After excluding duplicates and for other reasons, the remaining 2,997 articles were screened based on titles and abstracts. Subsequently, 2,584 articles were excluded as irrelevant, and the remaining 413 were subjected to a full-text review. Of these, 342 were excluded as non-randomized controlled trials, for incomplete data, as conference papers, or for non-compliance with intervention measures, among others reasons (Figure 4). Ultimately, 71 articles were included in the NMA (summarized in Tables 1–3).

**Table 1:** Characteristics of the included studies.

Characteristics	Mean	SD
Age	65.8	9.1
Exercise period (weeks)	14.5	15.3
Number of interventions (frequency)	2.5	1.05
Practice time (minutes)	54	24.7

**Table 2:** Relative effect sizes of efficacy on MDS-UPDRS-III according to network meta-analysis.

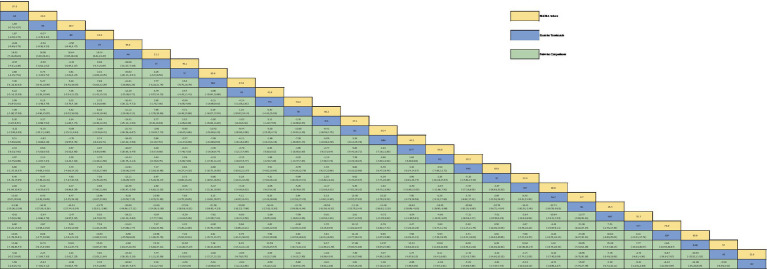

GE, Gait exercise; RC, Routine care; RE, Resistance Exercise; BE, Balance exercise; HT, hydrotherapy; AH, archery; ST, Stretching training; TC, shadowboxing; NW, Nordic walking; YG, yoga; TTC, treadmill training category; TB, treadmill Balance; DA, dance; SG, Sports game; MM, Mindfulness meditation; DTT, Dual task training; FQ, Fitness Qigong; HAS, High strength and agility; SC, Sport climbing; SST, Sports strategy training; Da2,dance2(Duality Rhythm Dance); Bo, Boxing; MIT, Moderate intensity training; STT, Sensory attention training; ESP, Elastic strap Pilates; RAB, Ride a bike; AE, Aerobic Exercise. The green box is pair-to-pair comparison, the blue box is intervention name, and the yellow box is SUCRA values.

**Table 3:** Characteristics of included trails in this network meta-analysis.

First author, year	Magazine (name)	Sample (I/C) (Mean age)	Hoehn-Yahr	Sample size (*n*)	Intervention	Details of interventions
Albrecht, 2021	Journal of Parkinson’s Disease	72	2–3	65	I: Gait exercises C: Routine care	1 h twice a week for 10 weeks
Bega, 2015	Yoga & Physical Therapy	67.3	1–3	14	I: Yoga C: Resistance exercise	For 12 weeks, 60 min twice a week
Biebl, 2022	Parkinson’s Disease	71.8	2–3	26	I: Balance exercise C: Gait exercises	6 weeks, 12 sessions, 30 min each
Bucken Gobbi, 2013	Motriz-Revista De Educacao Fisica	68.4	1–3	45	I: Resistance exercise/gait exercises C: Routine care	4 months, twice a week, 60 min each time
Cabrera-Martos, 2020	Clinical Rehabilitation	79	2–3	44	I: Balance exercise C: Routine care	8 weeks, three times a week, 45 min each time
Cancela, 2020	Rejuvenation Research	68.33	1–3	12	I: Resistance exercise C: Balance exercise	25 min once a week for 8 weeks
Canning, 2015	Neurology	70	2–3	231	I: Balance exercise C: Routine care	Six months, three times a week, 40-60 min each time
Carroll, 2017	Archives of Physical Medicine and Rehabilitation	71.42	1–3	21	I: Hydrotherapy C: Routine care	45 min twice a week for 6 weeks
Carvalho, 2015	Clinical Interventions in Aging	65.7	1–3	22	I: Gait exercises/routine care C: Resistance exercise	For 12 weeks, 30 min twice a week
Chen, 2023	Parkinson’s Disease	67.2	1–4	39	I: Archery C: Routine care	Once a week for 60 min for 12 weeks
Chen, 2021	Arquivos De Neuro-Psiquiatria	62.5	2–3	62	I: Resistance exercise/resistance exercise C: Stretching training	For 3 months, 50 min twice a week
Gao, 2014 (Gao et al., 2014)	Clinical Rehabilitation	76.8	1–4	76	I: Shadowboxing C: Routine care	60 min three times a week for 12 weeks
Collett, 2017	Journal of Neurology Neurosurgery and Psychiatry	65.5	1–2	89	I: Aerobic exercise C: Routine care	6 months, twice a week, 60 min each time
Corcos, 2013	Movement Disorders	58.6	1–3	48	I: Resistance exercise C: Routine care	For 24 months, twice a week for the first six months, once a week for the next six months
Cugusi, 2015	Neuro Rehabilitation	67.3	1–3	20	I: Nordic walking C: Routine care	60 min twice a week for 2 weeks
da Silva, 2023	Games for Health Journal	64	1–3	25	I: Hydrotherapy C: Routine care	For 10 weeks, 40 min twice a week
Elangovan, 2020	Sports Medicine and Health Science	67.0	1–3	18	I: Yoga C: Routine care	For 12 weeks, 60 min twice a week
Fisher, 2008	Archives of Physical Medicine & Rehabilitation	64	1–3	30	I: Treadmill training category C: Gait exercises/routine car	8 weeks, three times a week, 45 min each time
Freidle, 2022	NPJ Parkinson’s Disease	71.1	1–3	95	I: Balance exercise C: Routine care	10 weeks, three times a week, 60 min each time
Gandolfi, 2019	Movement Disorder	71.59	1–3	37	I: Balance exercise C: Routine care	4 weeks, twice a week, 60 min each time
Gassner, 2019	Journal of Parkinson’s Disease	67.6	2–3	38	I: Treadmill training balance C: Treadmill training category	8 weeks, twice a week, 30 min each time
Hackney, 2009	Journal of Rehabilitation Medicine	68.2	1–3	48	I: Dance/dance2 C: Routine care	1 h twice a week for 13 weeks
Hubble, 2018	American Journal of Physical Medicine & Rehabilitation	65.4	1–3	22	I: Balance exercise C: Routine care	Once a week for 12 weeks, 90 min
Jaggi, 2023	European Journal of Medical Research	72.4	1–4	40	I: Sports game C: Routine care	15 min five times a week
Kurt, 2018	Disability and Rehabilitation	63.2	2–3	40	I: Hydrotherapy C: Stretching training	5 weeks, 5 times a week, 60 min each time
Kwok, 2023	NPJ Parkinson’s Disease	64.5	1–3	68	I: Mindfulness meditation C: Stretching training	8 weeks, twice a week, 90 min each time
Kwok, 2019	Jama Neurology	63.7	1–3	138	I: Mindfulness meditation C: Stretching training	8 weeks, once a week, 90 min each time
Zhu, 2018	Clinical Rehabilitation	65.5	2–3	46	I: Hydrotherapy C: Dual-task training	6 weeks, 5 times a week, 30 min each time
Youm, 2020	Sensors (Basel, Switzerland)	69.7	1–3	17	I: Resistance exercise C: Routine care	12 weeks, three days a week, 90 min each time
Wayne, 2017	BMC Complementary and Alternative Medicine	62	2–2.5	25	I: Shadowboxing C: Routine care	60 min twice a week for 6 months
Santos, 2017	European Journal of Physical and Rehabilitation Medicine	73.38	1–2.5	28	I: Resistance exercise C: Routine care	8 weeks, twice a week, 60 min each time
Xiao, 2016	Geriatrics & Gerontology International	67.53	1–2.5	89	I: Fitness Qigong C: Gait exercises	45 min four times a week for six months
Wu, 2021	Japan Journal of Nursing Science	65.12	1–2	98	I: Aerobic Exercise C: Routine care	Eight weeks, three times a week for 50 min each time
Wang, 2022	Frontiers in Aging Neuroscience	67.65	1–2	45	I: Fitness Qigong C: Stretching training	24 weeks, three times a week, 90 min each time
Volpe, 2013	Movement Disorders	63.7	2–3	24	I: Dance C: Routine care	6 months, once a week, 90 min each time
vb6b, 2018	Medicine and Science in Sports and Exercise	64.2	2–3	39	I: Resistance exercise C: Balance exercise/routine care	Three months, twice a week for 50 min each time
van der Kolk, 2019	Lancet Neurology	59.4	1–2	130	I: Treadmill training category C: Stretching training	Six months, three times a week, 45 min each time
Tollar, 2019 (Tollar et al., 2019)	Medicine and Science in Sports and Exercise	67.6	1–3	55	I: Treadmill training category C: Routine care	For 2 years, 60 min three times a week
Tollar, 2018	Archives of Physical Medicine and Rehabilitation	67.6	2–3	55	I: High strength and agility C: Routine care	3 weeks, 5 times a week, 45 min each time
Solla, 2019	Journal of Alternative and Complementary Medicine	67.1	1–3	20	I: Dance C: Routine care	12 weeks, twice a week, 90 min each time
Soke, 2021	Acta neurologica Belgica	56.7	1–3	24	I: Dual-task training C: Aerobic exercise	For 8 weeks, 30 min three times a week
Silva-Batista, 2020	Movement Disorders	64.6	3–4	32	I: Balance exercise C: Stretching training	12 weeks, three times a week, 90 min each time
Landers, 2019	Movement Disorders	63.5	1–3	27	I: Treadmill training category C: Resistance exercise	Eight weeks, three times a week for 50 min each time
Langer, 2021 (Langer et al., 2021)	NPJ Parkinson’s Disease	64	2–3	46	I: Sport climbing C: Routine care	12 weeks, three times a week, 90 min each time
Li, 2012	New England Journal of Medicine	68	1–4	195	I: Shadowboxing C: Stretching training/resistance exercise	24 weeks, twice a week, 60 min each time
Li F, 2022	Frontiers in Human Neuroscience	67.7	1–3	51	I: Dance C: Routine care	4 weeks, 5 times a week, 60 min each time
Li X, 2022	Frontiers in Medicine	63.25	1–3	31	I: Dance C: Routine care	For 12 weeks, 5 times a week for 60 min each time
Li G, 2022	Translational Neurodegeneration	69.6	1–3	40	I: Fitness Qigong C: Routine care	12 weeks, twice a week, 90 min each time
Mak, 2021	Journal of Parkinson’s Disease	62.7	2–3	64	I: Gait exercises C: Resistance exercise	Six months, three times a week, every 90 min
Silva-Batista, 2016	Medicine and Science in Sports and Exercise	64.2	2–3	39	I: Balance exercise C: Resistance exercise/routine care	For 24 weeks, 50 min twice a week
Schlenstedt, 2015	Plos One	75.7	2–3	32	I: Resistance exercise C: Balance exercise	7 weeks, twice a week, 60 min each time
Nadeau, 2014	Medicine and Science in Sports and Exercise	61.95	1–2.5	34	I: Treadmill training category C: Routine care	24 weeks, 3 times a week, 60 min each time
Morris, 2017	Journal of Physiotherapy	71	1–4	120	I: Dual-task training C: Routine care	6 weeks, twice a week, 60 min each time
Morris, 2015	Neurorehabilitation and Neural Repair	68.4	1–4	195	I: Resistance exercise C: Sports strategy training/routine care	8 weeks, once a week, 2 h each time
Moratelli, 2022	Motriz: Revista de Educação Física	64.3	1–3	31	I: Duality rhythm dance C: Dance	12 weeks, twice a week, 45 min each time
Moon, 2020	Complementary Therapies in Clinical Practice	66.4	1–3	17	I: Fitness Qigong C: Routine care	For 12 weeks, 40 min three times a week
Sangarapillai, 2021	Neurorehabilitation and Neural Repair	64.	1–3	40	I: Boxing C: Stretching training	10 weeks, three times a week, 60 min each time
Romenets, 2015	Complementary Therapies in Medicine	63.2	1–3	33	I: Dance C: Routine care	For 12 weeks, 60 min twice a week
McKee, 2013	Journal of Motor Behavior	70.9	1–3	33	I: Dance C: Routine care	For 12 weeks, 20 min twice a week
Schenkman, 2018	Jama Neurology	63	1–2	128	I: Treadmill training category C: Moderate intensity training/routine care	26 weeks, four times a week, 50 min each time
Schenkman, 2012	Physical Therapy	63.4	1–3	121	I: Balance exercise C: Aerobic exercise/routine care	Four months, three times a week, 45 min each time
Sage, 2009	Movement Disorders	65.6	1–3	46	I: Sensory attention training C: Aerobic exercise/routine care	For 12 weeks, 60 min three times a week
Michels, 2018	Complementary Therapies in Medicine	70.9	1–3	13	I: Dance C: Routine care	10 weeks once a week, 60 min each time
Miyai, 2002	Archives of Physical Medicine & Rehabilitation	69.5	2–3	20	I: Treadmill training category C: Routine care	4 weeks, 3 times a week, 45 min each time
Mollinedo, 2018	Rejuvenation Research	66	1–3	26	I: Elastic strap Pilates C: Stretching training	For 12 weeks, 60 min twice a week
Millar, 2020	PloS One	67	1–3	18	I: Treadmill training category C: Moderate intensity training	10 weeks, three times a week, 60 min each time
Ni, 2016	Archives of Physical Medicine and Rehabilitation	72.2	1–3	37	I: Yoga C: Resistance exercise/routine care	For 12 weeks, 60 min twice a week
Pereira, 2022a	International Journal of Environmental Research and Public Health	68.13	1–4	15	I: Dual-task training C: Ride a bike	For 7 weeks, 20 min twice a week
Poliakoff, 2013	Neurorehabilitation	66.5	1–3	21	I: Treadmill training category C: Routine care	10 weeks, twice a week, 60 min each time
Jung, 2020	NPJ Parkinson’s Disease	69.2	1–5	82	I: Dual-task training C: Routine care	6 weeks, three times a week, 90 min each time
van Puymbroeck, 2018	Evidence-based Complementary and Alternative Medicine	50	1–2	37	I: Sports game C: Routine care	6 months, 60 min three times a week

### 4.2 Study characteristics

These 71 RCTs were published between 2002 and 2023, and involved a total of 3,732 participants. The NMA included a total of 87 intervention experiments and 27 distinct interventions: gait exercises (GE, such as gait posture interventions and aerobic walking), routine care: (RC), resistance exercises (RE, such as weightlifting, resistance band exercises, and strength training), balance exercises (BE, such as stability exercises and balance training), hydrotherapy (HT, aquatic exercises), archery (AH), stretching exercises (ST, such as limb and joint stretching), Tai Chi (TC), Nordic walking (NW), yoga(YG), treadmill training (TTC, high-intensity, moderate-intensity, or low-intensity), treadmill balance (TB, treadmill walking combined with balance interventions), dance (DA, including tango, Irish dance, improvised dance, waltz), sports games (VR, sports games), mindfulness meditation (MM), dual-task training (DTT), fitness qigong (FQ, Six-character Formula, Five Animal Frolics, Eight Brocades), high-intensity agility training (HAS), sport climbing(SC), sport strategy training(SST), dance 2 (Da2, binary rhythmic dance), boxing (BO), moderate-intensity training (MIT), sensory attention training (STT), elastic band Pilates (ESP), riding a bicycle (RAB), and aerobic exercise (AE). In the included studies, most exercise interventions were compared to routine care, stretching exercises, or aerobic exercises as the control. Among all eligible RCTs, 55 were two-arm (Albrecht et al., 2021; Bega et al., 2015; Biebl et al., 2022; Cabrera-Martos et al., 2020; Cancela et al., 2020; Canning et al., 2015; Carroll et al., 2017; Chen et al., 2023; Collett et al., 2017; Corcos et al., 2013; Cugusi et al., 2015; Da Silva et al., 2023; Elangovan et al., 2020; Freidle et al., 2022; Gandolfi et al., 2019; Gassner et al., 2019; Hubble et al., 2018; Jaggi et al., 2023; Jung et al., 2020; Kurt et al., 2018; Kwok et al., 2023; Kwok et al., 2019; Landers et al., 2016; Landers and Navalta, 2019; Li G. et al., 2022; Mak and Wong-Yu, 2021; McKee and Hackney, 2013; Michels et al., 2018; Millar, 2020; Miyai et al., 2002; Mollinedo-Cardalda et al., 2018; Moon et al., 2020; Moratelli et al., 2022; Morris et al., 2017; Ni et al., 2016; Pereira-Pedro et al., 2022a; Poliakoff et al., 2013; Romenets et al., 2015; Sangarapillai et al., 2021; Santos et al., 2017; Schlenstedt et al., 2015; Silva-Batista et al., 2020; Soke et al., 2021; Solla et al., 2019; van der Kolk et al., 2019; Van Puymbroeck et al., 2018; Volpe et al., 2013; Wang et al., 2022; Wayne et al., 2017; Wu et al., 2021; Xiao and Zhuang, 2016; Youm et al., 2020; Zhu et al., 2018; vb6b, 2018) and 16 were three-arm (Bucken Gobbi et al., 2013; Carvalho et al., 2015; Chen et al., 2021; Fisher et al., 2008; Hackney and Earhart, 2009; Li et al., 2012; Li F. et al., 2022; Li X. et al., 2022; Morris et al., 2015; Nadeau et al., 2014; Sage and Almeida, 2009; Schenkman et al., 2012; Schenkman et al., 2018; Silva-Batista et al., 2016; Tollar et al., 2018). The exercise intervention period for included trials ranged from 4 to 96 weeks (average 14.5 weeks, SD 15.3 weeks), the frequency of exercise intervention from 1 to 5 sessions per week (average 2.5, SD 1.05), and the total time per session from 15 to 120 min (average 54 min, SD 24.7 min).

### 4.3 Quality assessment

Methodological quality assessment results for the eligible RCTs are depicted in Figure 1. While overall quality was high, 8 trials did not mention random sequence generation or blinding, and one trial reported incomplete results. These trials were classified as “medium risk”. Additionally, 23 trials mentioned randomization and blinding but did not provide specific details. These trials were classified as “low risk”.

### 4.4 Network meta-analysis for efficacy ranking

Figure 2A depicts the network diagram of different exercise interventions for the MDS-UPDRS-III. The overall network structure indicates numerous comparisons between routine care (control) and dance, stretching training, balance exercise, and resistance exercises, as these interventions are currently popular. Also indicated are numerous pair-wise comparisons between interventions from three-arm trials. Surface Under the Cumulative Ranking (SUCRA) curves for each of the 27 intervention type are shown in Figure 2B (derived from Table 2). In these curves (red lines), a sharp early increase yields a larger area and indicates a greater probability of improving motor ability (higher efficacy rank), whilst a shallow, later increase yields a smaller area and is indicative of lower probability of motor improvement (lower efficacy rank). According to these analyses, archery ranked first (SUCRA = 95.6%), followed by bike riding (SUCRA = 80.9%), duality rhythm dance (SUCRA = 80.8%), elastic strap Pilates (SUCRA = 76.9%), sensory attention training (SUCRA = 70.7%), treadmill balance training (SUCRA = 70.2%), yoga (SUCRA = 67.8%), high-intensity strength and agility training (SUCRA = 67.2%), resistance exercise (SUCRA = 40.2%), and balance exercise (SUCRA = 39.7%). The efficacies of these exercise interventions for reducing MDS-UPDRS-III score were higher than stretching training (SUCRA = 21.1%) and routine care (SUCRA = 22.6%).

Further, archery was significantly superior to routine care (standardized mean difference (SMD = −16.92, 95%CI = −28.97, −4.87), stretch training (SMD = −19.08, 95%CI = −31.07, −7.08), sports games (SMD = −21.73, 95%CI = −36.58, −6.87), and aerobic exercise (SMD = −14.33, 5%CI = −26.50, −2.16) for improving motor abilities in Parkinson’s disease. Overall, boxing was the least effective as MDS-UPDRS-III score was not reduced post-intervention (SUCRA = 0.7%, Sangarapillai et al., 2021). All other interventions were superior to boxing.

### 4.5 Efficacy ranking

The cumulative ranking probability according to SUCRA graphs was as follows: Archery > Ride a bike > Duality Rhythm Dance > Elastic strap Pilates > Sensory attention training > Treadmill > Balance > Yoga > High strength and agility > Nordic walking > Dance > Sport climbing > Aerobic Exercise > Fitness Qigong > Shadowboxing > Dual task training > Sports strategy training > Treadmill training category > Resistance Exercise > Balance exercise > Gait exercise > Mindfulness meditation > Moderate intensity training > Routine care > Hydrotherapy > Stretching training > Sports game > Boxing.

### 4.6 Consistency analysis

The global inconsistency analysis *p*-value was 0.2170, indicating no significant inconsistency. Additionally, the node-splitting model analysis yielded *p*-values >0.05, indicating no significant inconsistency between direct and indirect comparisons, supporting adoption of a consistency model.

### 4.7 Publication bias

Publication bias for the outcome measure (MDS-UPDRS-III) was further evaluated by constructing a funnel plot with relative effect size (odds ratio, OR) on the horizontal axis and standard error of log (OR) on the vertical axis, and then examining plot dispersion and symmetry (Figure 3). This contrast yields narrower, higher plots for studies with larger sample sizes and lower, more dispersed plots for studies with smaller sample sizes. The majority of points falling within the 95% confidence intervals (slash lines) is indicative of little or no heterogeneity, while a symmetrical distribution is indicative of little or no publication bias. The points representing individual comparisons (indicated by color code in the lower panel) fell mainly within the 95%CIs and with high symmetry on each side of the 0 point (no effect), suggesting little publication bias.

## 5 Discussion

The objective of this NMA was to integrate evidence from 71 RCTs (including 87 interventions and 27 different exercises) to identify those with greatest efficacy for improving the motor abilities of PD patients according to MDS-UPDRS-III score reduction. Surface Under the Cumulative Ranking curve analysis indicated that archery (Chen et al., 2023; Radder et al., 2020) is the most effective intervention for reducing MDS-UPDRS-III scores and improving motor abilities (Chen et al., 2023; Radder et al., 2020), surpassing the efficacy of all other exercises tested (SUCRA = 95.6%), followed by bicycling (Pereira-Pedro et al., 2022b) and duality rhythm dance. This particular dance form, characterized by binary rhythm movements distinct from traditional dance categories such as tango, waltz, Irish dance, and self-created free dance, proved surprisingly effective (SUCRA = 80.8%), providing clues to the precise activity patterns (e.g., muscle groups engaged and contraction–relaxation dynamics) most beneficial for improving motor abilities in PD. Previous studies have reported significant improvements in motor abilities following exercise interventions such as dance, dual-task training, and high-intensity resistance training (Wang et al., 2023; Zhou et al., 2022), and these interventions were also relatively effective according to the current NMA. However, many previous meta-analyses and reviews grouped distinct exercise interventions into a single category, such as “martial arts” for Tai Chi, fitness Qigong, or boxing (Radder et al., 2020). Although this grouping increased statistical power, it did not identify the best specific intervention, and as demonstrated here, there were marked differences in therapeutic efficacy among these interventions. The current analysis thus provides precise information for selecting the most appropriate exercise intervention.

Archery has long been regarded an ideal rehabilitative activity, and was one of the first exercises introduced for the rehabilitation of paralysis and limb palsy patients (Guttmann and Mehra, 1973). Archery involves the activation of trunk latissimus dorsi and serratus anterior muscles, along with the stretching of the palm, finger muscles, and wrist. Participants must mentally focus on specifically ordered steps, from hooking the bowstring with their fingers to releasing the arrow by activating and relaxing various muscle groups in precise sequences, thereby providing opportunities for both strength and coordination enhancement. The practice of archery also provides a definitive metric for success (target hits), thus motivating regular participation (regular upper limb functional exercises) and performance improvement. Indeed, regular archery is reported to improve overall body stability and even non-motor symptoms (Chen et al., 2023). However, there have been a limited number of RCTs applying archery as an intervention for PD, so additional trials are required to confirm these findings. Further, as compliance will improve efficacy, additional studies are needed to compare the effects of exercises matched for weekly frequency, intensity, and total duration.

Riding a bike, the second most effective exercise choice for PD patients (SUCRA = 80.9%), can improve cardiovascular health, motor skills, coping, and cognitive skills as well as provide a sense of independence and promote social inclusion (Alberts et al., 2011; Ridgel et al., 2013; Tiihonen et al., 2021). Low-intensity progressive cycling improved motor dysfunction (Chang et al., 2018) while high-intensity cycling improved motor function, stiffness, and bradykinesia by promoting activity-dependent neuroplasticity (Feng et al., 2020; Oliveira de Carvalho et al., 2018). Forced passive cycling was also reported to enhance functional connectivity between the motor cortex and ipsilateral thalamus and between the subthalamic nucleus and posterior cingulate (Shah et al., 2016), consistent with improvements in motor control via neuroplasticity within sensorimotor pathways. Moreover, the motor improvements conferred by regular cycling may involve enhanced processing of proprioceptive inputs by sensory cortex (Nagano-Saito et al., 2005).

Dance is another exercise intervention widely used as therapy for PD patients, and consistent with previous reports of improved motor abilities, the SUCRA value was among the highest (80.8%). The benefits of dance likely stem from the multifaceted nature of the activity, requiring movement control (fluidity) and appropriate posture, potentially addressing PD-related deficits such as stiffness, bradykinesia, and postural instability (Hashimoto et al., 2015; Sharp and Hewitt, 2014; Šumec et al., 2015). Dance is also highly enjoyable, aiding in compliance (Earhart, 2009). At the neural level, dance stimulates basal ganglia circuits and reward systems to evoke positive emotions (Weintraub et al., 2005).

This NMA has several limitations. First, it included only early- to mid-stage PD patients (average Hoehn-Yahr stage of 1–3), so results may not be applicable to more advanced PD patients. There was also substantial heterogeneity in the frequency and duration of exercise interventions across trials, which could influence efficacy independent of the specific exercise used. Third, despite a comprehensive search, all included studies were in English, which may introduce culture bias against other exercise practices. Many pair-wise comparisons also included only a few individual trials, limiting the statistical power. Although all participants included in the analysis were in the early and middle stages of PD, the MDS-UPDRS-III scores varied markedly, likely reflecting the subjective nature of the assessment and inter-rater variability.

Last, most studies did not report concealed allocation, which may result in selection and performance biases. Large-scale RCTs comparing multiple exercise modalities matched for intensity and duration, and with appropriate safeguards against bias are needed to confirm the rankings presented here.

## 6 Conclusion

To the best of our knowledge, this study is the first to compare a large number of distinct exercise modalities (*n* = 27) for efficacy in improving motor function among patients with early- to middle-stage PD. A series of direct and indirect comparisons using NMA and SUCRA methods identified archery, cycling and dual rhythm dance as particularly effective for improving MDS-UPDRS-III scores, while others such as boxing and sports gameplay were largely ineffective. Although larger-sample, multi-arm trials are required for validation, the current findings may serve as a useful guide for healthcare providers when selecting exercise interventions to enhance the motor abilities, quality of life, and cardiovascular health status of individuals with PD.

## Data Availability

The original contributions presented in the study are included in the article/supplementary material, further inquiries can be directed to the corresponding author.

## Glossary

GE: Gait exercise
RC: Routine care
RE: Resistance exercise
BE: Balance exercise
HT: hydrotherapy
AH: Archery
ST: Stretching training
TC: Shadowboxing
NW: Nordic walking
YG: Yoga
TTC: Treadmill training category
TB: Treadmill Balance
DA: Dance
SG: Sports game
MM: Mindfulness meditation
DTT: Dual task training
FQ: Fitness Qigong
HAS: High strength and agility
SC: Sport climbing
SST: Sports strategy training
DA2: Dance2
BO: Boxing
MIT: Moderate intensity training
STT: Sensory attention training
ESP: Elastic strap Pilates
RAB: Ride a bike
AE: Aerobic Exercise
